# Molecular characterization of *Plasmodium falciparum* antifolate resistance markers in Thailand between 2008 and 2016

**DOI:** 10.1186/s12936-020-03176-x

**Published:** 2020-03-04

**Authors:** Rungniran Sugaram, Kanokon Suwannasin, Chanon Kunasol, Vivek Bhakta Mathema, Nicholas P. J. Day, Prayuth Sudathip, Preecha Prempree, Arjen M. Dondorp, Mallika Imwong

**Affiliations:** 1 Department of Molecular Tropical Medicine and Genetics, Faculty of Tropical Medicine, Mahidol University, 420/6 Rajvithi Rd., Bangkok, 10400 Thailand; 2 Division of Vector Borne Diseases, Department of Disease Control, Ministry of Public Health, Nonthaburi, Thailand; 3 Mahidol-Oxford Tropical Medicine Research Unit, Faculty of Tropical Medicine, Mahidol University, Bangkok, Thailand; 4 Centre for Tropical Medicine, Churchill Hospital, Oxford, UK

**Keywords:** *pfdhfr*, *pfdhps*, *pfgch1*, Sulphadoxine–pyrimethamine

## Abstract

**Background:**

Resistance to anti-malarials is a major threat to the control and elimination of malaria. Sulfadoxine–pyrimethamine (SP) anti-malarial treatment was used as a national policy for treatment of uncomplicated falciparum malaria in Thailand from 1973 to 1990. In order to determine whether withdrawal of this antifolate drug has led to restoration of SP sensitivity, the prevalence of genetic markers of SP resistance was assessed in historical Thai samples.

**Methods:**

*Plasmodium falciparum* DNA was collected from the Thailand–Myanmar, Thailand–Malaysia and Thailand–Cambodia borders during 2008–2016 (N = 233). Semi-nested PCR and nucleotide sequencing were used to assess mutations in *Plasmodium falciparum dihydrofolate reductase* (*pfdhfr*), *P. falciparum dihydropteroate synthase* (*pfdhps*). Gene amplification of *Plasmodium falcipaurm* GTP cyclohydrolase-1 (*pfgch1*) was assessed by quantitative real-time PCR. The association between *pfdhfr*/*pfdhps* mutations and *pfgch1* copy numbers were evaluated.

**Results:**

Mutations in *pfdhfr*/*pfdhsp* and *pfgch1* copy number fluctuated overtime through the study period. Altogether, 14 unique *pfdhfr*–*pdfhps* haplotypes collectively containing quadruple to octuple mutations were identified. High variation in *pfdhfr*–*pfdhps* haplotypes and a high proportion of *pfgch1* multiple copy number (51% (73/146)) were observed on the Thailand–Myanmar border compared to other parts of Thailand. Overall, the prevalence of septuple mutations was observed for *pfdhfr*–*pfdhps* haplotypes. In particular, the prevalence of *pfdhfr*–*pfdhps*, septuple mutation was observed in the Thailand–Myanmar (50%, 73/146) and Thailand–Cambodia (65%, 26/40) border. In Thailand–Malaysia border, majority of the *pfdhfr*–*pfdhps* haplotypes transaction from quadruple (90%, 9/10) to quintuple (65%, 24/37) during 2008–2016. Within the *pfdhfr*–*pfdhps* haplotypes, during 2008–2013 the *pfdhps* A/S436F mutation was observed only in Thailand–Myanmar border (9%, 10/107), while it was not identified later. In general, significant correlation was observed between the prevalence of *pfdhfr* I164L (ϕ = 0.213, *p*-value = 0.001) or *pfdhps* K540E/N (ϕ = 0.399, *p*-value ≤ 0.001) mutations and *pfgch1* gene amplification.

**Conclusions:**

Despite withdrawal of SP as anti-malarial treatment for 17 years, the border regions of Thailand continue to display high prevalence of antifolate and anti-sulfonamide resistance markers in falciparum malaria. Significant association between *pfgch1* amplification and *pfdhfr* (I164L) or *pfdhps* (K540E) resistance markers were observed, suggesting a compensatory mutation.

## Background

Both falciparum and vivax malaria remains an important public health problem in border regions of Thailand. Resistance to anti-malarials presents a major hurdle for eradication of the disease [1]. Drug resistance in both *Plasmodium falciparum* and *Plasmodium vivax* has been reported as early as the 1950s [2]. Sulfadoxine–pyrimethamine (SP), a folate pathway inhibitor was deployed in Thailand for the treatment of uncomplicated falciparum malaria from 1973 until 1991 [3]. By 1991, substantial loss of the SP drug efficacy prompted a change in first-line treatment in Thailand [3]. Molecular investigations revealed that mutations in *P. falciparum* dihydrofolate reductase (*pfdhfr*) and *P. falciparum* dihydropteroate synthase (*pfdhps*) were associated with SP treatment failures and could be used as molecular markers for SP resistance [4, 5]. The mutations in *pfdhfr* and *pfdhps* often occurred in a step-wise progressive manner resulting in increased levels of drug resistance [4, 6]. Resistance to antifolates has also been linked to gene amplification of *P. falciparum* guanosine triphosphate cyclohydrolase 1 (*pfgch1*)-an enzyme responsible for coding a crucial enzyme in the folate pathway [7]. Parasites with *pfgch1* amplification were reportedly less susceptible to antifolates as elevated expression of enzymes assisted antifolate resistance by competing with the drugs [7], and compensating the loss of fitness caused by mutations in *pfdhfr* and *pfdhps* by increasing the flux of metabolic products in the folate pathway [8]. An earlier study from Thailand reported a high proportion of parasites carrying multiple copies of *pfgch1* and suggested an association between *pf*g*ch1* copy number variation (CNV) and the *pfdhfr* (I164L) mutations [8]. Several studies conducted between 1995 and 2008 have identified varying levels of triple or quadruple mutations in *pfdhfr* and *pfdhps* [5, 8, 9]. A more recent survey conducted in Ubonratchathani province close the Thailand–Cambodia borders, which had a lot of reports in many anti-malarial drug resistances [2, 3], showed high levels of *pfdhfr* (N51I, C59R, and S108N, ≥ 76%) and *pfdhps* (A437G, K540E, A581G or A437G, K540N, A581G or S436A, A437G, K540E, ≥ 90%) triple mutations [10]. These border areas are malaria endemic regions. Each site is geographically distant from other and often experiences high migration of diverse human population. However, data on the current status of antifolate and anti-sulfonamide resistance markers in *P. falciparum* in other major border regions of Thailand is scarce. Presumably, the persistence of highly mutations on SP-resistant markers related to the using of other drugs that may also induced pressure on *pfdhfr* and *pfdhps* of malaria parasite. The trimethoprim–sulfamethoxazole, which is used to treat acute respiratory infections, presented cross-resistance with pyrimethamine and sulfadoxine [11, 12]. Reemergence of chloroquine-sensitive *P. falciparum* in Malawi after a decade-long cessation of drug use shows that for some anti-malarials restoration of drug sensitivity is possible after removal of the drug pressure [13]. However, several factors including drug target, nature of genes and host/parasite genetic background may differently affect the persistence of SP resistance after removal of SP use.

The present study is a retrospective molecular surveillance of three antifolate and anti-sulfonamide resistance markers in samples obtained from the malaria endemic border provinces of Thailand between 2008 and 2016. It aimed at describing the current status of resistance markers after long-term cessation of SP as an anti-malarial treatment in Thailand.

## Methods

### Study sites and samples collection

Between 2008 and 2016, dried blood spots samples were collected using Whatman 3MM CHR filter paper from *P. falciparum* malaria patients in nine provinces bordering Thailand (Maehongson N = 24; Tak N = 16; Kanchanaburi N = 70; Ranong N = 34; Suratthani N = 2; Ubonratchathani N = 9; Sisaket N = 19; Trat N = 12; and Yala N = 47 (Additional file 1: Table S3). All samples were acquired during anti-malarial drug therapeutic efficacy monitoring projects that were approved by the Research Ethics Committee of the Department of Disease Control, Ministry of Public Health, and Faculty of Tropical Medicine, Mahidol University, Thailand. DNA was extracted using QIAamp DNA Mini Kit (QIAGEN, Germany) and stored at 4 °C before analysis.

### *Pfdhfr* and *pfdhps* sequencing and quality control

The *pfdhfr* and *pfdhps* genes were amplified using semi-nested PCR as described previously [14–17] (Additional file 1: Table S1). Amplified final products were sequenced to assess *pfdhfr* (A16V, C50R, N51I, C59R, S108N and I164L) and *pfdhfr* (A/S436F, A437G, K540E/N, A581G and A613S/T) mutations. The initial semi-nested PCR generated products measured 880 bp for *pfdhfr* and 715 bp for *pfdhps*. The nested PCR products were purified by using FavorPrep GEL/PCR Purification Kit (FAVORGEN^®^BIOTECH CORP., Taiwan) and sequenced by Macrogen Inc. (South Korea). The sequencing results were analysed using BioEdit software version 7.2.6. Resistance marker sequences were compared with reference sequences for *pfdhfrs* (DHFR-TS PF3D7_0417200) and *pfdhsp* (PPPK-DHPS PF3D7_0810800).

### *Plasmodium falciparum gch1* copy number estimation

To assess *pfgch1* amplification, quantitative real-time PCR (qPCR) of the *pfgch1* gene was compared with a single copy reference gene (*seryl* tRNA *ligase*) (Accession No: A0A0L1I323). The specific probes and primers used have been described previously [8] (Additional file 1: Table S1). A laboratory *pfgch1* single copy strain and *pfgch1* multiple copies strain of *P. falciparum* were used as reference standard. The copy number of *pfgch1* gene was analysed from the threshold cycle (C_t_) and ΔΔC_t_ values calculated as follows: ΔΔC_t_ = (C_t_ of *pfgch1* − C_t_ of *pfSerRs*) of sample − (C_t_ of *pfgch1* − C_t_ of *pfSerRs*) of Calibrator. The copy number of *pfgch1* were subsequently calculated using 2^−ΔΔCt^ method [18]. The *pfgch1* amplification cut off value was set at 1.5 copies number.

### Statistical analysis

The *pfdhfr*/*pfdhps* mutations and *pfgch1* copy numbers were described as the proportion of each haplotype present at each border region. The changing trend of antifolate resistance markers after drug withdrawal was analysed by comparing the prevalence of markers over time by year. Chi-square (χ^2^) and the non-parametric phi correlation coefficient statistics (ϕ) was used to evaluate the difference in frequency and associations respectively among the group of each antifolate resistance marker. A *p*-value ≤ 0.05 was considered statistically significant.

## Results

### Mutations in *pfdhfr* and *pfdhps* genes in isolates from the Thailand–Myanmar border

**Fig. 1:**
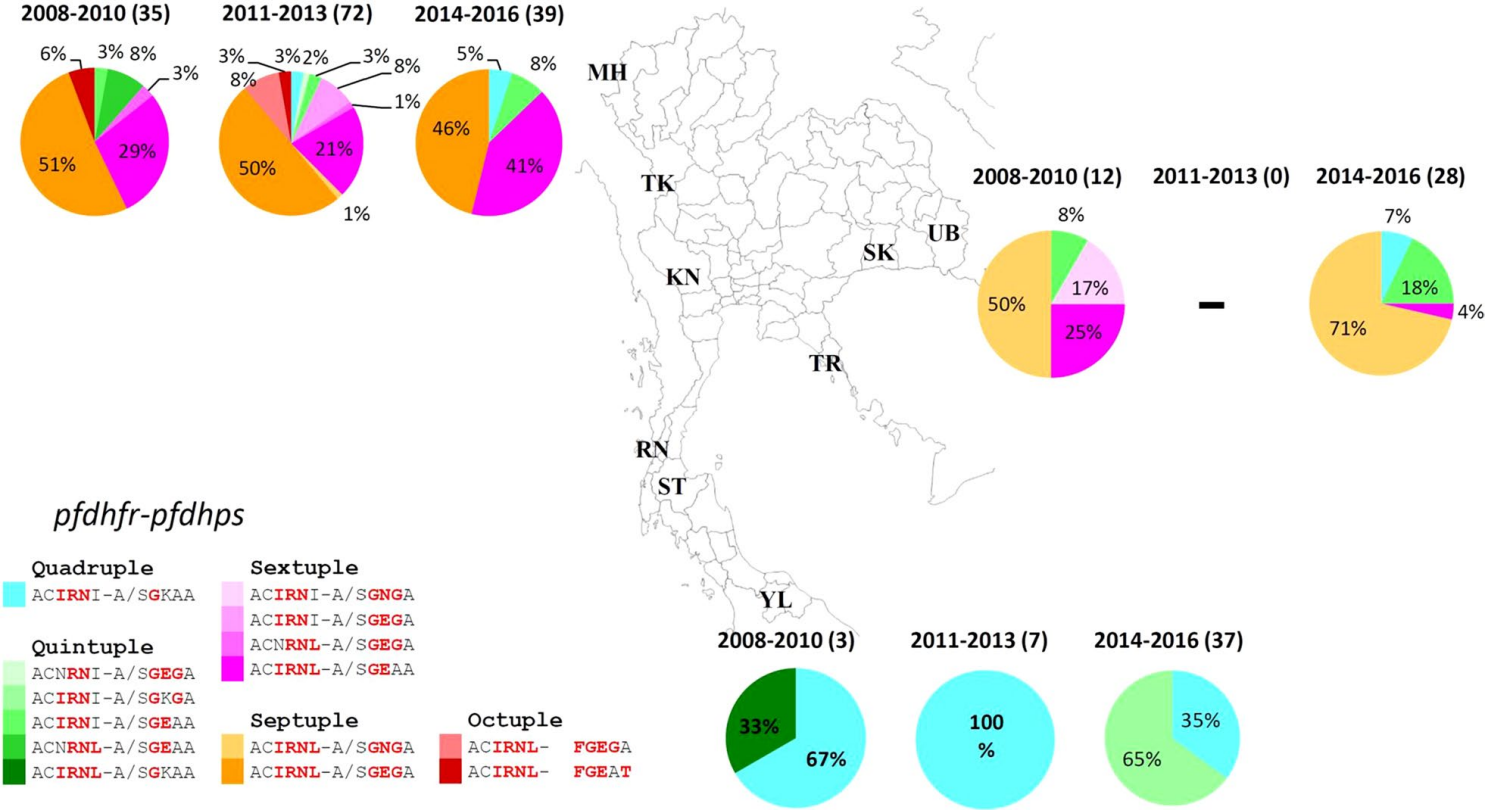
Prevalence of SP resistance markers *pfdhfr*, *pfdhps*, and *pfgch1* in *P. falciparum* obtained from malaria patients between 2008 and 2016. (*TH* Thailand, *MY* Myanmar, *CH* Cambodia, *ML* Malaysia, *MH* Maehongson Province, *TK* Tak Province, *KN* Kanchanaburi Province, *RN* Ranong Province, *ST* Suratthani Province, *YL* Yala Province, *TR* Trat Province, *SK* Srisaket Province, *UB* Ubonratchathani Province)

A total of 146 *P. falciparum* samples collected from malaria clinics in five provinces between 2008 and 2016 were analysed (Table 1). The six point mutations of *pfdhfr* gene and five point mutations of *pfdhps* associated with resistance were assessed in all samples. Septuple mutations at N51I, C59R, S108N and I164L of *pfdhfr* with A437G, K450E and A581G of *pfdhps* presented as the main haplotype that was found in 51% (18/35), 50% (36/72) and 46% (18/39) from samples between 2008 and 2010, 2011–2013 and 2014–2016, respectively. The secondary group of the main haplotype was the parasite containing sextuple mutations at N51I, C59R, S108N and I164L for *pfdhfr* with A437G and K450E mutation in *pfdhps* that showed 29% (10/35), 21% (15/72) and 41% (16/39), respectively. However, the highly resistance falciparum parasites were the Octuple haplotype which consisted of the mutation at N51I, C59R, S108N and I164L for *pfdhfr* and A437G, K450E, A581G and A/S436F or A613S/T for *pfdhps* were detected only in 2008–2010 (6%, 2/35) and 2011–2013 (3%, 2/72) (Fig. 1).

**Table 1:** The *pfdhfr/pfdhps* haplotypes and *pfgch1* copy number variation of *P. falciparum* separated by Thailand-neighboring countries between 2008 and 2016

Area	Year^a^	DHFR						DHPS					GCH1	Num.
		A16*V*	C50*R*	N51*I*	C59*R*	S108*N*	I164*L*	A/S436*F*	A437*G*	K540*E*/*N*	A581*G*	A613*S*/*T*	*S*ingle/*M*ultiple	
Thailand-Myanmar border	2008–2010	A	C	N	*R*	*N*	*L*	A/S	*G*	*E*	A	A	S	3
		A	C	N	*R*	*N*	*L*	A/S	*G*	*E*	*G*	A	S	1
		A	C	*I*	*R*	*N*	I	A/S	*G*	*E*	A	A	*M*	1
		A	C	*I*	*R*	*N*	*L*	A/S	*G*	*E*	A	A	S	4
		A	C	*I*	*R*	*N*	*L*	A/S	*G*	*E*	A	A	*M*	6
		A	C	*I*	*R*	*N*	*L*	A/S	*G*	*E*	*G*	A	S	12
		A	C	*I*	*R*	*N*	*L*	A/S	*G*	*E*	*G*	A	*M*	6
		A	C	*I*	*R*	*N*	*L*	*F*	*G*	*E*	A	*T*	S	2
	2011–2013	A	C	N	*R*	*N*	I	A/S	*G*	*E*	*G*	A	*M*	1
		A	C	N	*R*	*N*	*L*	A/S	*G*	*E*	*G*	A	S	1
		A	C	*I*	*R*	*N*	I	A/S	*G*	*E*	A	A	S	1
		A	C	*I*	*R*	*N*	I	A/S	*G*	*E*	A	A	*M*	1
		A	C	*I*	*R*	*N*	I	A/S	*G*	*E*	*G*	A	S	1
		A	C	*I*	*R*	*N*	I	A/S	*G*	*E*	*G*	A	*M*	5
		A	C	*I*	*R*	*N*	I	A/S	*G*	*K*	A	A	S	2
		A	C	*I*	*R*	*N*	*L*	A/S	*G*	*E*	A	A	S	11
		A	C	*I*	*R*	*N*	*L*	A/S	*G*	*E*	A	A	*M*	4
		A	C	*I*	*R*	*N*	*L*	A/S	*G*	*E*	*G*	A	S	19
		A	C	*I*	*R*	*N*	*L*	A/S	*G*	*E*	*G*	A	*M*	17
		A	C	*I*	*R*	*N*	*L*	A/S	*G*	*N*	G	A	*M*	1
		A	C	*I*	*R*	*N*	*L*	*F*	*G*	*E*	A	*T*	S	1
		A	C	*I*	*R*	*N*	*L*	*F*	*G*	*E*	A	*T*	*M*	1
		A	C	*I*	*R*	*N*	*L*	*F*	*G*	*E*	*G*	A	S	1
		A	C	*I*	*R*	*N*	*L*	*F*	*G*	*E*	*G*	A	*M*	5
	2014–2016	A	C	*I*	*R*	*N*	I	A/S	*G*	*E*	A	A	S	2
		A	C	*I*	*R*	*N*	I	A/S	*G*	*E*	A	A	*M*	1
		A	C	*I*	*R*	*N*	I	A/S	*G*	K	A	A	S	2
		A	C	*I*	*R*	*N*	*L*	A/S	*G*	*E*	A	A	S	4
		A	C	*I*	*R*	*N*	*L*	A/S	*G*	*E*	A	A	*M*	12
		A	C	*I*	*R*	*N*	*L*	A/S	*G*	*E*	*G*	A	S	5
		A	C	*I*	*R*	*N*	*L*	A/S	*G*	*E*	*G*	A	*M*	10
Total														146
Thailand–Cambodia border	2008–2010	A	C	*I*	*R*	*N*	I	A/S	*G*	*E*	A	A	S	1
		A	C	*I*	*R*	*N*	I	A/S	*G*	*N*	*G*	A	S	2
		A	C	*I*	*R*	*N*	*L*	A/S	*G*	*E*	A	A	S	2
		A	C	*I*	*R*	*N*	*L*	A/S	*G*	*E*	A	A	*M*	1
		A	C	*I*	*R*	*N*	*L*	A/S	*G*	*N*	*G*	A	S	6
	2014–2016	A	C	*I*	*R*	*N*	I	A/S	*G*	*E*	A	A	S	5
		A	C	*I*	*R*	*N*	I	A/S	*G*	K	A	A	S	1
		A	C	*I*	*R*	*N*	I	A/S	*G*	K	A	A	*M*	1
		A	C	*I*	*R*	*N*	*L*	A/S	*G*	*E*	A	A	S	1
		A	C	*I*	*R*	*N*	*L*	A/S	*G*	*N*	*G*	A	S	19
		A	C	*I*	*R*	*N*	*L*	A/S	*G*	*N*	*G*	A	*M*	1
Total														40
Thailand–Malaysia border	2008–2010	A	C	*I*	*R*	*N*	I	A/S	*G*	K	A	A	S	2
		A	C	*I*	*R*	*N*	*L*	A/S	*G*	K	A	A	S	1
	2011–2013	A	C	*I*	*R*	*N*	I	A/S	*G*	K	A	A	S	7
	2014–2016	A	C	*I*	*R*	*N*	I	A/S	*G*	K	A	A	S	13
		A	C	*I*	*R*	*N*	I	A/S	*G*	K	*G*	A	*M*	5
		A	C	*I*	*R*	*N*	I	A/S	*G*	K	*G*	A	S	19
Total														47

Each italic upper script letters represent mutation observed in the amino acid at the corresponding position

^a^The mutations observed are grouped based on year of sample collection

**Fig. 2:**
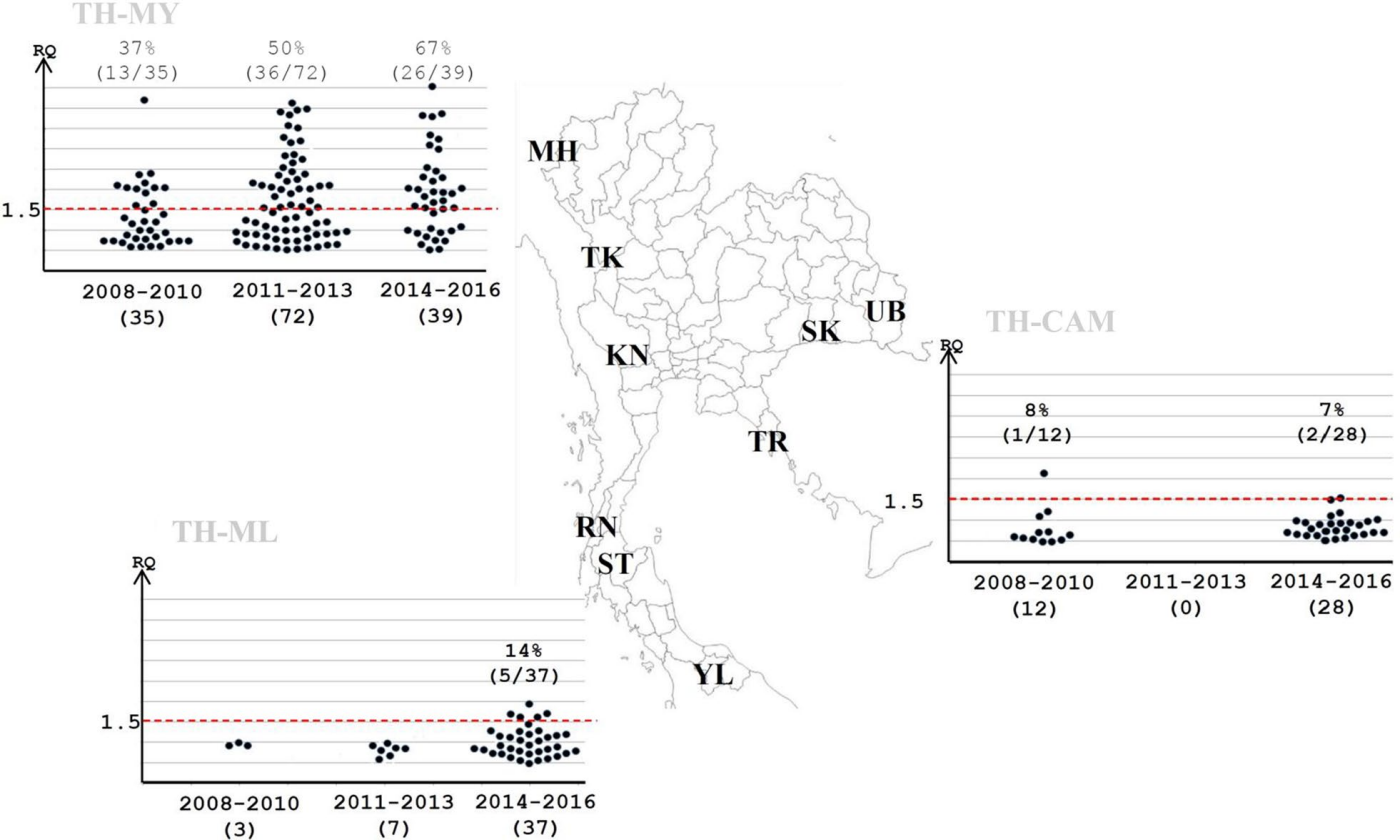
The *pfgch1* copy number variation in *P. falciparum* obtained from malaria patients between 2008 and 2016. (*TH* Thailand, *MY* Myanmar, *CH* Cambodia, *ML* Malaysia, *MH* Maehongson Province, *TK* Tak Province, *KN* Kanchanaburi Province, *RN* Ranong Province, *ST* Suratthani Province, *YL* Yala Province, *TR* Trat Province, *SK* Srisaket Province, *UB* Ubonratchathani Province)

The proportion of *P. falciparum* samples with *pfgch1* gene amplification from the Thailand–Myanmar border increased from 37% (13/35) in year 2008 to 67% (26/39) in year 2016 (Fig. 2).

### Mutations in *pfdhfr* and *pfdhps* genes in isolates from the Thailand–Cambodia border

*Plasmodium falciparum* isolates (N = 40) from malaria clinics in three provinces along Thailand–Cambodia border were collected between 2008 and 2016 (Table 1). Major haplotype was the septuple mutants that comprised of mutations at N51I, C59R, S108N and I164L in *pfdhfr* gene and A437G, K450N and A581G in *pfdhps* gene. The *P. falciparum* samples with septuple mutation *pfdhfr*–*pfdhps* were detected 50% (6/12) in 2008–2010 and 71% (20/28) in 2014–2016. The secondary haplotype in 2008–2010 was sextuple mutations haplotype that was composed of two haplotypes, namely, ACIRNL-A/SGEAA (25% (3/12)) and ACIRNI-A/SGNGA (17% (2/12)) then the prevalence of falciparum parasites with ACIRNL-A/SGEAA decreased to 4% (1/28) in 2014–2016. Whereas, prevalence of falciparum parasite with quintuple mutations (ACIRNI-A/SGEAA) increased from 8% (1/12) in 2008–2010 to 18% (5/28) in 2014–2016 (Fig. 1). The multiple copy number of *pfgch1* gene were observed in 7% (2/28)–8% (1/12) in this region (Fig. 2).

### Mutations in *pfdhfr* and *pfdhps* genes in isolates from the Thailand–Malaysia border

All 47 *P. falciparum* isolates from Yala province represented the samples from Thailand–Malaysia border (Table 1). All falciparum samples carried quadruple or quintuple mutation of *pfdhfr*–*pfdhps* gene. The major haplotype had mutations at N51I, C59R and S108N of *pfdhfr* and A437G of *pfdhps* that showed 67% (2/3) in 2008–2010, 100% (7/7) in 2011–2013 and 35% (13/37) in 2014–2016. Quintuple mutations (ACIRNI-A/SGKGA) haplotype became the main haplotype in the last period of this study (65%, 24/37) (Fig. 1). In addition, *pfgch1* gene amplification was found in 14% (5/37) of genes in 2014–2016 in contrast to absence of multiple copies in earlier samples (Fig. 2).

**Fig. 3:**
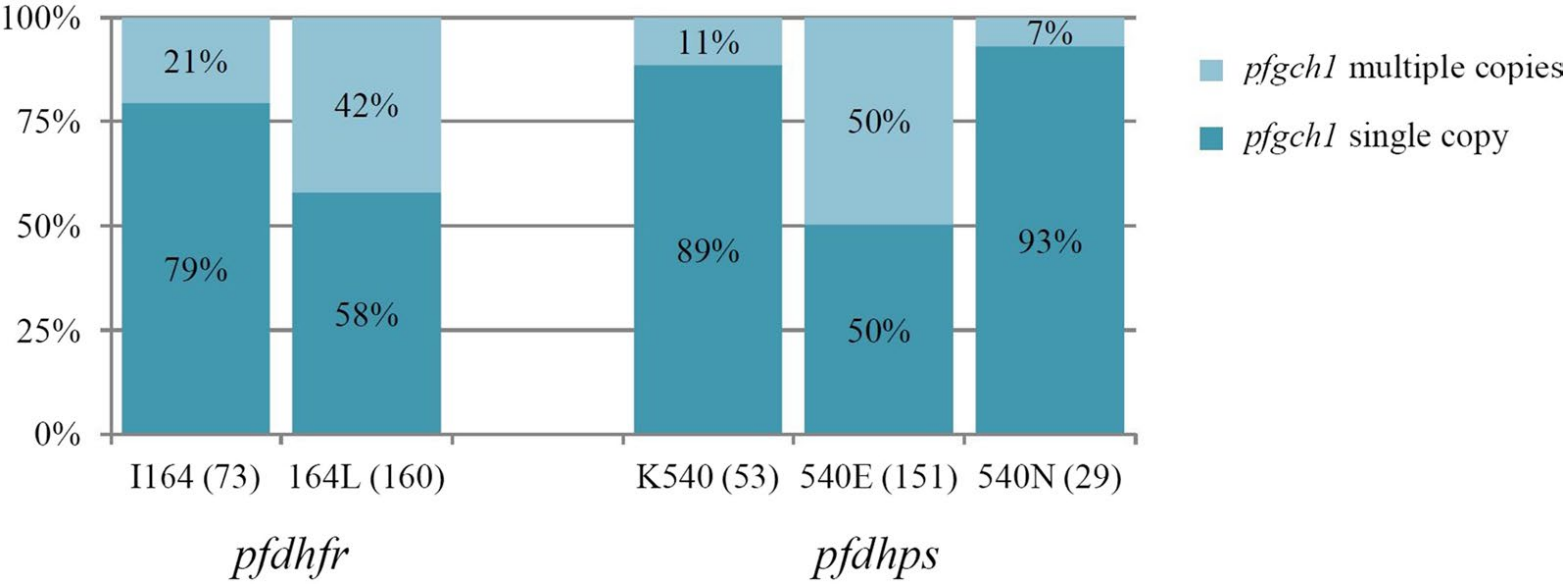
Prevalence of *pfdhfr* codon I164L, *pfdhps* codon K540E/N and *pfgch1* copy number in *P. falciparum* in Thailand between 2008 and 2016. *pfdhfr*: *Plasmodium falciparum dihydrofolate reductase*; *pfdhps*: *P. falciparum dihydropteroate synthase*

Comparing frequencies of mutations from three areas, prevalence of septuple (ACIRNL-A/SGE/NGA) and sextuple mutations (ACIRNL-A/SGEAA, ACNRNL-A/SGEGA and ACIRNI-A/SGE/NGA) were found in the falciparum samples from Thailand–Myanmar and Thailand–Cambodia border as the major group. Whereas, the falciparum samples of Thailand–Malaysia border presented only quintuple and quadruple mutations haplotypes. The octuple mutations which had the mutation at A/S436F or A613S/T were detected only in Thailand–Myanmar border. However, both of octuple haplotype observed below 8% in 2008–2013 were absented in 2014–2016 (Fig. 3).

### Associations between *pfgch1* copy number and antifolate resistance markers in *pfdhfr* and *pfdhps*

The association between individual six mutation points in *pfdhfr* and gene amplification of *pfgch1* were assessed in all 233 samples. Only the I164L *pfdhfr* point mutation was significantly associated with *pfgch1* amplification (ϕ = 0.213, *p*-value= 0.001). *Plasmodium falciparum* carrying both the *pfdhfr* I164 allele and *pfgch1* multiple copies were observed in only 21% (15/73) of samples, while *P. falciparum* isolates that carried both *pfdhfr* 164L and *pfgch1* amplification were observed in 42% (67/160) of samples (Fig. 3).

Similarly, evaluation of the association between five mutation points in *pfdhps* and *pfgch1* gene amplification showed that the *pfdhps* K540E/N allele was significantly correlated to *pfgch1* amplification (ϕ = 0.399, *p*-value ≤ 0.001). *Plasmodium falciparum* carrying the *pfdhps* 540E allele and *pfgch1* multiple copies was observed in 50% (75/151) of samples.

## Discussion

This longitudinal study of antifolate resistance markers in Thailand *P. falciparum* infections showed persisting high prevalence of antifolate resistance haplotypes between 2008 and 2016, despite long-term cessation of SP treatment regimen. Studies from early 1990s to 2014, revealed the *pfdhfr* point mutations transitioning from double mutations (S108N with N51I/C59R/164L, ≥ 72%) to triple (N51I, C59R, S108N or C59R, S108N, I164L) and eventually quadruple (N51I, C59R, S108N/T, I164L) mutations in provinces of Thailand bordering Myanmar and Cambodia [5, 10, 19–21]. Present findings are in agreement with previously observed trends showing that most *P. falciparum* parasites up to 75% carried triple or quadruple mutations in *pfdhfr*. However, limited data from 1994 to 1995 on the Thailand–Malaysia border indicated a high prevalence of 88% *pfdhfr* triple mutations, whereas no quadruple mutations were reported [22].

The *pfdhps* mutations based on four codons (S436F/A, A437G, A581G, and A613S/T) were first reported in mid 1990s along the Thailand–Myanmar border [5]. Multiple studies conducted between 2001 and 2007 collectively indicated fluctuating dominance of the *pfdhps* triple (2001–2003, > 95%), quadruple (2002–2003, > 67%) and triple (2004–2007, > 97%) mutations [9, 19, 23]. Taken together, previous reports [9, 10] and present findings indicate emerging trend in shift from *pfdhps* double to triple mutations as major mutant group along the Thailand–Myanmar and Thailand–Cambodia borders.

Resistance level to antifolate drug in falciparum malaria was increased and conferred by mutations in *dhfr* and *dhps* gene. The combination of *pfdhfr* N51I, C59R and S108N and *pfdhps* A437G and K540E/N (quintuple mutant) strongly predicts clinical outcome [24]. The mutation in *pfdhfr* I164L and *pfdhps* A581G and A613S/T developed later and were associated with increase in antifolate resistance [6].

Effect of mutations in *pfdhfr* on the inhibition constant (K_i_) for pyrimethamine has been well described [25–28]. This suggests that quadruple mutations including the 164 position were critically important for the development of resistance to pyrimethamine. In addition, it was shown that K_i_ for sulfadoxine differed significantly between wild type and triple mutant parasites carrying K540E mutation [4, 29]. Strong association between elevated copy number of the *pfgch1* gene and the *pfdhfr*-I164L mutation has been shown previously [8]. In this study, an association between *pfgch1* gene multiple copy number and the *pfdhfr*-I164L mutation was also observed. However, in Ghana with low level of *pfgch1* gene amplification (6%; 12/192), no *dhfr*-I164L was detected in samples with amplified *pfgch1* [30]. The positive correlation between *pfdhps* (540E) and *pfgch1* multiple copies observed in this study was not observed before. These correlations in parasites with high-level antifolate and anti-sulfonamide resistance may indicate functional linkage and fitness epistasis between genes on different chromosomes. The observed positive correlations between *pfgch1* amplification and *pfdhfr* (I164L)/*pfdhps* (K540E/N) mutations may represent adaptation of the parasite dissociated from the anti-malarial drug pressure or arguably the pressure from non-anti-malarial antifolate drugs.

Present study provides indication of co-increase in quintuple mutants and *pfgch1* multiple copies in five isolates during 2014–2016. However, relatively smaller sample size (in total 37 isolates) from Thailand–Malaysia border might also have influenced the observed results. Persistence of high prevalence of antifolate resistance haplotypes in Thailand may be explained by several factors. Persistence of antifolate resistance markers might result from continued drug pressure from non-malarial antifolate drugs such as trimethoprim and sulfamethoxazole [11]. Trimethoprim–sulfamethoxazole (*a.k.a* Bactrim™) is still used in Thailand [31] as a part of standard package to care people with HIV/AIDS [32], urinary tract infection [33], melioidosis [34] and pneumonia [35]. The antifolate gene mutations might have been sustained because of continued presence of this antifolate drug pressure [11].

Human migration contributed to the spread of chloroquine resistant malaria from Southeast Asia to Africa during the 1970s, and SP resistance during the 1980s–1990s [36–38]. Studies conducted during 1998–2001 [39] and 2007–2009 [40], identified a high proportion of *pfdhfr/pfdhps* resistance haplotypes in parts of Myanmar sharing border regions with Thailand. This period coincided with the increased prevalence of *pfdhfr/pfdhps* mutants in Thailand [9, 19, 20]. The main haplotypes then were quadruple mutants for *pfdhfr* and triple mutants for *pfdhps*, respectively (Additional file 1: Table S2). The Thailand–Cambodia border showed a slight difference in the proportion of *pfdhfr* resistance haplotypes containing quadruple mutations, which was higher than the proportion of triple mutations observed between 2003 and 2007 [21], whereas the prevalence of *pfdhps* mutations was similar to Cambodia [20] (Additional file 1: Table S2). Present findings are in agreement with the low reported *pfdhfr*–*pfdhps* mutations on the Thailand–Malaysia border compared to other regions [9, 22]. It is likely that the parasite population in this border area, which is a conflict zone, is separate from the other border areas, with limited human migration carrying parasites between these areas.

## Conclusions

Decades after cessation of SP as anti-malarial treatment in Thailand, there is a persistent high proportion of *P. falciparum* carrying both sulfadoxine and pyrimethamine resistance markers, with the exception of the Thailand–Malaysia border. Amplification of *pfgch1* correlated with *pfdhfr* (I164L) and *pfdhps* (K540E) mutations, suggesting *pfgch1* amplification might be compensatory to mutations in the *pfdhfr* and *pfdhps* genes.

## Supplementary information

**Additional file 1: Table S1.** Primer and Probe sequences for detection of SP resistance markers in *P. falciparum*. **Table S2.** The prevalence reports of *pfdhfr* and *pfdhps* mutations close the border of Thailand and neighboring countries since 1990 to 2016. **Table S3.** The *Pfdhfr/Pfdhps* haplotypes and *gch1* copy number variation of *P. falciparum* in 9 provinces along the border of Thailand-neighboring countries between 2008 and 2016.

## Data Availability

The dataset generated during the current study are available from corresponding author on reasonable request.

## Abbreviations

*pfdhfr*: *Plasmodium falciparum dihydrofolate reductase*
*pfdhps*: *P. falciparum dihydropteroate synthase*
*pfgch1*: *P. falciparum GTP cyclohydrolase-1*
SNPs: Single nucleotide polymorphisms
CNV: Copy number variation
