# Supplementary material for: Molecular characterization of Plasmodium falciparum antifolate resistance markers in Thailand between 2008 and 2016
Source: Malar J. 2020 Mar 4;19:107. doi: 10.1186/s12936-020-03176-x (PMC7055081; doi:10.1186/s12936-020-03176-x)
Supplement: Supplementary file 1 — Additional file 1: Table S1. Primer and Probe sequences for detection of SP resistance markers in P. falciparum. Table S2. The prevalence reports of pfdhfr and pfdhps mutations close the border of Thailand and neighboring countries since 1990 to 2016. Table S3. The Pfdhfr/Pfdhps haplotypes and gch1 copy number variation of P. falciparum in 9 provinces along the border of Thailand-neighboring countries between 2008 and 2016. [file 12936_2020_3176_MOESM1_ESM.docx]

**Molecular characterization of *Plasmodium falciparum* antifolate resistance markers in Thailand between 2008 and 2016**

**Supplementary material**

**Table S1** Primer and Probe sequences for detection of SP resistance markers in *P. falciparum*.

| Detections | Primers/Probes | Sequence (5’-3’) | Reference |
| --- | --- | --- | --- |
| Nest1-*pfdhfr* | *pfdhfr*_PrF | ATGGARSAMSTYTSMGABGTWTTY  [ATGGA(G/A)(G/C)A(A/C)(G/C)T(T/C)T(G/C)(A/C)GA(G/T/C)GT(A/T)TTY] | [14, 16] |
|  | *pfdhfr*_M5 | AGTATATACATCGCTAACAGA | [14] |
| Nest2-*pfdhfr* | *pfdhfr*_PrNF | TSMGABGTWTTYGAYATTTAYGC  [T(G/C)(A/C)GA(G/T/C)GT(A/T)TT(T/C)GA(T/C)ATTTA(T/C)GC] | [14, 17] |
|  | *pfdhfr*_M5 | AGTATATACATCGCTAACAGA | [14] |
| Nest1-*pfdhps* | *pfdhps*_F | GTTGAACCTAAACGTGCTGT | [14, 15] |
|  | *pfdhps*_R/ | AATTGTGTGATTTGTCCACAA | [14] |
| Nest2-*pfdhps* | *pfdhps*_R2 | AACCTAAACGTGCTGTTCAA | [14] |
|  | *pfdhps*_R/ | AATTGTGTGATTTGTCCACAA | [14] |
| *gch1* copy number | *pfgch1*_F | CCTTTTGAAGGTACATGTGATATTGAGT | [8] |
|  | *pfgch1*_R | GCGTTACAAATATCGTTAGTTAAATCTTCT | [8] |
|  | *pfSerRs*_F | TTAGATTTTCAAGCGAGACGTTTAAA | [8] |
|  | *pfSerRs*_R | CCTTCCTACGGCTAAACCTGAAC | [8] |
|  | *pfgch1*_Probe | CTTGAAAATTTAGATAACCCGA | [8] |
|  | *pfSerRs*_Probe | CCAATAATTTCTGCCATACTA | [8] |

**Table S2** The prevalence reports of *pfdhfr* and *pfdhps* mutations close the border of Thailand and neighboring countries since 1990 to 2016

| Years | Main mutations prevalence | | | | | | | | | | | |
| --- | --- | --- | --- | --- | --- | --- | --- | --- | --- | --- | --- | --- |
|  | Thailand-Myanmar | | | | Thailand-Cambodia | | | | Thailand-Malaysia | | | |
|  | Thailand | | Myanmar | | Thailand | | Cambodia | | Thailand | | Malaysia | |
|  | *pfdhfr* | *pfdhps* | *pfdhfr* | *pfdhps* | *pfdhfr* | *pfdhps* | *pfdhfr* | *pfdhps* | *pfdhfr* | *pfdhps* | *pfdhfr* | *pfdhps* |
| 1990 |  |  |  |  | 3Ms: 25.00% 4Ms: 75.00% [21] |  |  |  |  |  |  |  |
| 1991 |  |  |  |  |  |  |  |  |  |  |  |  |
| 1992 |  |  |  |  |  |  |  |  |  |  |  |  |
| 1993 |  |  |  |  |  |  |  |  |  |  |  |  |
| 1994 |  |  |  |  |  |  |  |  |  |  | 2Ms: 12.50% 3Ms: 87.50% [22] | 1M: 7.69%  2Ms: 81.25% [22] |
| 1995 |  |  |  |  |  |  |  |  |  |  |  |  |
| 1996 |  |  |  |  |  |  |  |  |  |  |  |  |
| 1997 |  |  |  |  |  |  |  |  |  |  |  |  |
| 1998 |  |  | 3Ms: 22.09% 4Ms: 71.78% [39] | 2Ms: 11.46%  3Ms: 88.54% [39] |  |  |  |  |  |  |  |  |
| 1999 |  |  |  |  |  |  |  |  |  |  |  |  |
| 2000 |  |  |  |  |  |  |  |  |  |  |  |  |
| 2001 | 3Ms: 14.00% 4Ms: 86.00% [20] | 1M: 1.28%  3Ms: 97.44% [20] |  |  |  |  |  |  |  |  |  |  |
| 2002 |  |  |  |  |  |  |  |  |  |  |  |  |
| 2003 | 3Ms: 20.83% 4Ms: 68.75% [19] |  |  |  | 3Ms: 37.50% 4Ms:62.50% [21] |  |  |  |  |  |  |  |
| 2004 |  |  |  |  |  |  | 3Ms: 54.29% 4Ms: 40.00% [20] | 3Ms: 86.11% 2Ms: 8.33% [20] |  |  |  |  |
| 2005 |  |  |  |  |  |  |  |  |  |  |  |  |
| 2006 |  |  |  |  |  |  |  |  |  |  |  |  |
| 2007 |  | 2Ms: 1.80% 3Ms: 96.70%  [9] | 3Ms: 43.59% 4Ms: 50.43% [40] | 3Ms: 81.20% 4Ms: 8.55% [40] | 3Ms: 23.81% 4Ms:76.19% [21] | 2Ms:21.05% 3Ms:76.32% [9] |  |  |  | 2Ms:100.00% [9] |  |  |
| 2008 |  |  |  |  |  |  |  |  |  |  |  |  |
| 2009 |  |  |  |  |  |  |  |  |  |  |  |  |
| 2010 |  |  |  |  |  |  |  | 2Ms: 26.89% 3Ms: 50.94% [17] |  |  |  |  |
| 2011 |  |  |  |  |  |  |  |  |  |  |  |  |
| 2012 |  |  |  |  |  |  |  |  |  |  |  |  |
| 2013 |  |  |  |  |  |  |  |  |  |  |  |  |
| 2014 |  |  |  |  | 3Ms: 78.72% 4Ms:21.28% [10] | 3Ms: 97.87% 4Ms:2.13% [10] |  |  |  |  |  |  |
| 2015 |  |  |  |  |  |  |  |  |  |  |  |  |
| 2016 |  |  |  |  |  |  |  |  |  |  |  |  |

1M: single mutation, 2Ms: Double mutations, 3Ms: Triple mutations, 4Ms: Quadruple mutations

**Table S3**. The *Pfdhfr/Pfdhps* haplotypes and *gch1* copy number variation of *P. falciparum* in 9 provinces along the border of Thailand- neighboring countries between 2008 and 2016.

| Border Area | Provinces | Year | DHFR | | | | | | DHPS | | | | | GCH1 | Number | Percentage | Total |
| --- | --- | --- | --- | --- | --- | --- | --- | --- | --- | --- | --- | --- | --- | --- | --- | --- | --- |
|  |  |  | A16**V** | C50**R** | N51**I** | C59**R** | S108**N** | I164**L** | A/S436**F** | A437**G** | K540**E/N** | A581**G** | A613**S/T** | Single 0.5-1.5 |  |  |  |
| Thailand-Myanmar | RANONG | 2008-2010 | A | C | N | **R** | **N** | **L** | A/S | **G** | **E** | A | A | S | 1 | 8 |  |
|  | RANONG | 2008-2010 | A | C | **I** | **R** | **N** | I | A/S | **G** | **E** | A | A | M | 1 | 8 |  |
|  | RANONG | 2008-2010 | A | C | **I** | **R** | **N** | **L** | A/S | **G** | **E** | A | A | S | 4 | 31 |  |
|  | RANONG | 2008-2010 | A | C | **I** | **R** | **N** | **L** | A/S | **G** | **E** | A | A | M | 2 | 15 |  |
|  | RANONG | 2008-2010 | A | C | **I** | **R** | **N** | **L** | A/S | **G** | **E** | **G** | A | S | 5 | 38 | 13 |
|  | RANONG | 2011-2013 | A | C | **I** | **R** | **N** | I | A/S | **G** | K | A | A | S | 2 | 12 |  |
|  | RANONG | 2011-2013 | A | C | **I** | **R** | **N** | I | A/S | **G** | **E** | A | A | S | 1 | 6 |  |
|  | RANONG | 2011-2013 | A | C | **I** | **R** | **N** | I | A/S | **G** | **E** | A | A | M | 1 | 6 |  |
|  | RANONG | 2011-2013 | A | C | **I** | **R** | **N** | **L** | A/S | **G** | **E** | A | A | S | 7 | 41 |  |
|  | RANONG | 2011-2013 | A | C | **I** | **R** | **N** | **L** | A/S | **G** | **E** | A | A | M | 1 | 6 |  |
|  | RANONG | 2011-2013 | A | C | **I** | **R** | **N** | **L** | A/S | **G** | **E** | **G** | A | S | 3 | 18 |  |
|  | RANONG | 2011-2013 | A | C | **I** | **R** | **N** | **L** | A/S | **G** | **E** | **G** | A | M | 2 | 12 | 17 |
|  | RANONG | 2014-2016 | A | C | **I** | **R** | **N** | **L** | A/S | **G** | **E** | A | A | S | 1 | 25 |  |
|  | RANONG | 2014-2016 | A | C | **I** | **R** | **N** | **L** | A/S | **G** | **E** | **G** | A | M | 3 | 75 | 4 |
|  | KANCHANABURI | 2008-2010 | A | C | **I** | **R** | **N** | **L** | A/S | **G** | **E** | A | A | M | 4 | 33 |  |
|  | KANCHANABURI | 2008-2010 | A | C | **I** | **R** | **N** | **L** | A/S | **G** | **E** | **G** | A | S | 4 | 33 |  |
|  | KANCHANABURI | 2008-2010 | A | C | **I** | **R** | **N** | **L** | A/S | **G** | **E** | **G** | A | M | 2 | 17 |  |
|  | KANCHANABURI | 2008-2010 | A | C | **I** | **R** | **N** | **L** | **F** | **G** | **E** | A | **T** | S | 2 | 17 | 12 |
|  | KANCHANABURI | 2011-2013 | A | C | **I** | **R** | **N** | I | A/S | **G** | **E** | **G** | A | S | 1 | 4 |  |
|  | KANCHANABURI | 2011-2013 | A | C | **I** | **R** | **N** | I | A/S | **G** | **E** | **G** | A | M | 1 | 4 |  |
|  | KANCHANABURI | 2011-2013 | A | C | **I** | **R** | **N** | **L** | A/S | **G** | **E** | A | A | S | 4 | 15 |  |
|  | KANCHANABURI | 2011-2013 | A | C | **I** | **R** | **N** | **L** | A/S | **G** | **E** | A | A | M | 3 | 11 |  |
|  | KANCHANABURI | 2011-2013 | A | C | **I** | **R** | **N** | **L** | A/S | **G** | **E** | **G** | A | S | 8 | 30 |  |
|  | KANCHANABURI | 2011-2013 | A | C | **I** | **R** | **N** | **L** | A/S | **G** | **E** | **G** | A | M | 8 | 30 |  |
|  | KANCHANABURI | 2011-2013 | A | C | **I** | **R** | **N** | **L** | **F** | **G** | **E** | A | **T** | S | 1 | 4 |  |
|  | KANCHANABURI | 2011-2013 | A | C | **I** | **R** | **N** | **L** | **F** | **G** | **E** | A | **T** | M | 1 | 4 | 27 |
|  | KANCHANABURI | 2014-2016 | A | C | **I** | **R** | **N** | I | A/S | **G** | **E** | A | A | S | 2 | 6 |  |
|  | KANCHANABURI | 2014-2016 | A | C | **I** | **R** | **N** | I | A/S | **G** | **E** | A | A | M | 1 | 3 |  |
|  | KANCHANABURI | 2014-2016 | A | C | **I** | **R** | **N** | **L** | A/S | **G** | **E** | A | A | S | 3 | 10 |  |
|  | KANCHANABURI | 2014-2016 | A | C | **I** | **R** | **N** | **L** | A/S | **G** | **E** | A | A | M | 12 | 39 |  |
|  | KANCHANABURI | 2014-2016 | A | C | **I** | **R** | **N** | **L** | A/S | **G** | **E** | **G** | A | S | 4 | 13 |  |
|  | KANCHANABURI | 2014-2016 | A | C | **I** | **R** | **N** | **L** | A/S | **G** | **E** | **G** | A | M | 9 | 29 | 31 |
|  | MAE HONG SON | 2008-2010 | A | C | N | **R** | **N** | **L** | A/S | **G** | **E** | A | A | S | 2 | 20 |  |
|  | MAE HONG SON | 2008-2010 | A | C | N | **R** | **N** | **L** | A/S | **G** | **E** | **G** | A | S | 1 | 10 |  |
|  | MAE HONG SON | 2008-2010 | A | C | **I** | **R** | **N** | **L** | A/S | **G** | **E** | **G** | A | S | 3 | 30 |  |
|  | MAE HONG SON | 2008-2010 | A | C | **I** | **R** | **N** | **L** | A/S | **G** | **E** | **G** | A | M | 4 | 40 | 10 |
|  | MAE HONG SON | 2011-2013 | A | C | N | **R** | **N** | I | A/S | **G** | **E** | **G** | A | M | 1 | 7 |  |
|  | MAE HONG SON | 2011-2013 | A | C | N | **R** | **N** | **L** | A/S | **G** | **E** | **G** | A | S | 1 | 7 |  |
|  | MAE HONG SON | 2011-2013 | A | C | **I** | **R** | **N** | **L** | A/S | **G** | **E** | **G** | A | S | 2 | 14 |  |
|  | MAE HONG SON | 2011-2013 | A | C | **I** | **R** | **N** | **L** | A/S | **G** | **E** | **G** | A | M | 4 | 29 |  |
|  | MAE HONG SON | 2011-2013 | A | C | **I** | **R** | **N** | **L** | **F** | **G** | **E** | **G** | A | S | 1 | 7 |  |
|  | MAE HONG SON | 2011-2013 | A | C | **I** | **R** | **N** | **L** | **F** | **G** | **E** | **G** | A | M | 5 | 36 | 14 |
|  | TAK | 2011-2013 | A | C | **I** | **R** | **N** | I | A/S | **G** | **E** | **G** | A | M | 4 | 29 |  |
|  | TAK | 2011-2013 | A | C | **I** | **R** | **N** | **L** | A/S | **G** | **E** | **G** | A | S | 6 | 43 |  |
|  | TAK | 2011-2013 | A | C | **I** | **R** | **N** | **L** | A/S | **G** | **E** | **G** | A | M | 3 | 21 |  |
|  | TAK | 2011-2013 | A | C | **I** | **R** | **N** | **L** | A/S | **G** | **N** | **G** | A | M | 1 | 7 | 14 |
|  | TAK | 2014-2016 | A | C | **I** | **R** | **N** | **L** | A/S | **G** | **E** | **G** | A | S | 1 | 50 |  |
|  | TAK | 2014-2016 | A | C | **I** | **R** | **N** | **L** | A/S | **G** | **E** | **G** | A | M | 1 | 50 | 2 |
|  | SURAT THANI | 2014-2016 | A | C | **I** | **R** | **N** | I | A/S | **G** | K | A | A | S | 2 |  | 2 |
| Thailand-Cambodia | TRAT | 2011-2013 | A | C | **I** | **R** | **N** | I | A/S | **G** | **E** | A | A | S | 1 | 8 |  |
|  | TRAT | 2011-2013 | A | C | **I** | **R** | **N** | I | A/S | **G** | **N** | **G** | A | S | 2 | 17 |  |
|  | TRAT | 2011-2013 | A | C | **I** | **R** | **N** | **L** | A/S | **G** | **E** | A | A | S | 2 | 17 |  |
|  | TRAT | 2011-2013 | A | C | **I** | **R** | **N** | **L** | A/S | **G** | **E** | A | A | M | 1 | 8 |  |
|  | TRAT | 2011-2013 | A | C | **I** | **R** | **N** | **L** | A/S | **G** | **N** | **G** | A | S | 6 | 50 | 12 |
|  | SISAKET | 2014-2016 | A | C | **I** | **R** | **N** | I | A/S | **G** | K | A | A | S | 1 | 5 |  |
|  | SISAKET | 2014-2016 | A | C | **I** | **R** | **N** | I | A/S | **G** | K | A | A | M | 1 | 5 |  |
|  | SISAKET | 2014-2016 | A | C | **I** | **R** | **N** | I | A/S | **G** | **E** | A | A | S | 1 | 5 |  |
|  | SISAKET | 2014-2016 | A | C | **I** | **R** | **N** | **L** | A/S | **G** | **E** | A | A | S | 1 | 5 |  |
|  | SISAKET | 2014-2016 | A | C | **I** | **R** | **N** | **L** | A/S | **G** | **N** | **G** | A | S | 14 | 74 |  |
|  | SISAKET | 2014-2016 | A | C | **I** | **R** | **N** | **L** | A/S | **G** | **N** | **G** | A | M | 1 | 5 | 19 |
|  | UBONRATCHATHANI | 2014-2016 | A | C | **I** | **R** | **N** | I | A/S | **G** | **E** | A | A | S | 4 | 44 |  |
|  | UBONRATCHATHANI | 2014-2016 | A | C | **I** | **R** | **N** | **L** | A/S | **G** | **N** | **G** | A | S | 5 | 56 | 9 |
| Thailand-Malaysia | YALA | 2008-2010 | A | C | **I** | **R** | **N** | I | A/S | **G** | K | A | A | S | 2 | 67 |  |
|  | YALA | 2008-2010 | A | C | **I** | **R** | **N** | **L** | A/S | **G** | K | A | A | S | 1 | 33 | 3 |
|  | YALA | 2011-2013 | A | C | **I** | **R** | **N** | I | A/S | **G** | K | A | A | S | 7 | 100 | 7 |
|  | YALA | 2014-2016 | A | C | **I** | **R** | **N** | I | A/S | **G** | K | A | A | S | 13 | 35 |  |
|  | YALA | 2014-2016 | A | C | **I** | **R** | **N** | I | A/S | **G** | K | **G** | A | S | 19 | 51 |  |
|  | YALA | 2014-2016 | A | C | **I** | **R** | **N** | I | A/S | **G** | K | **G** | A | M | 5 | 14 | 37 |
| TOTAL | | | | | | | | | | | | | | | | | 233 |
